# The Galvanic Poultice

**Published:** 1851-05

**Authors:** 


*The Galvanic Poultice.*—M. Recamier has proposed a new method of employing galvanism locally, to which the above term may be fairly applied. The "poultice" is a small mass of cotton wad, containing a layer of thin zinc plates, and another layer of copper plates. The wad is enclosed in a small bag, one surface of which is defended by some waterproof tissue. The poultice is applied as closely as possible to the skin, the impermeable surface being external. The cutaneous perspiration soon accumulates in the interior of the wad, and, being of an acid character, gives rise to the developement of a galvanic current between the zinc, and copper plates.—*London Medical Times, April 12th,* 1851.

